# Transfemoral Transcatheter Aortic Valve Implantation at Hospitals Without On-Site Cardiac Surgery (TAVI at Home): A Multicenter Prospective Interventional Study

**DOI:** 10.3390/jcdd12020063

**Published:** 2025-02-10

**Authors:** Miriam Compagnone, Gianni Dall’Ara, Simone Grotti, Greta Mambelli, Elisabetta Fabbri, Carlo Savini, Marco Balducelli, Andrea Santarelli, Elia Iorio, Beatriz Vaquerizo, Alfredo Marchese, Giuseppe Tarantini, Francesco Saia, Chiara Zingaretti, Carolina Moretti, Caterina Cavazza, Bernadette Vertogen, Filippo Ottani, Andrea Rubboli, Oriana Nanni, Carmine Pizzi, Marcello Galvani, Fabio Felice Tarantino

**Affiliations:** 1 Interventional and Structural Cardiology Unit Forlì-Cesena, 47121 Forlì, Italy; 2 Department of Medical and Surgical Sciences—DIMEC—Alma Mater Studiorum, University of Bologna, 40138 Bologna, Italy; 3 IRCCS Istituto Romagnolo per lo Studio dei Tumori (IRST) “Dino Amadori”, 47014 Meldola, Italy; 4 U.O. Ricerca Valutativa e Policy dei Servizi Sanitari, AUSL Romagna, 48121 Ravenna, Italy; 5 GVM Care & Research Maria Cecilia Hospital, 48033 Cotignola, Italy; 6 Division of Cardiology, S. Maria delle Croci Hospital, Ravenna, AUSL Romagna, 48121 Ravenna, Italy; 7 Cardiology Unit, Bufalini Hospital, 47521 Cesena, Italy; 8 UOC Cardiologia e UTIC, Ente Ecclesiastico Ospedale Generale Regionale F Miulli, Acquaviva delle Fonti, 70021 Bari, Italy; 9 Hospital del Mar, 08003 Barcelona, Spain; 10 Ospedale S. Maria GVM Care & Research, 70124 Bari, Italy; 11 Department of Cardiac-Thoracic-Vascular Sciences and Public Health, University of Padova, 35128 Padua, Italy; 12 Interventional Cardiology Unit, IRCCS University Hospital of Bologna, Policlinico S. Orsola, 40138 Bologna, Italy; 13 Cardiovascular Department, Infermi Hospital, AUSL Romagna, 47921 Rimini, Italy; 14 Cardiology Division, Morgagni Pierantoni University Hospital, AUSL Romagna, 47121 Forlì, Italy; 15 Cardiovascular Research Unit, Fondazione Cardiologica Sacco, 47100 Forlì, Italy

**Keywords:** aortic valve stenosis, transcatheter aortic valve implantation, heart team

## Abstract

Transcatheter aortic valve implantation (TAVI) has become the standard of care for elderly patients with aortic stenosis. International guidelines recommend that TAVI should be performed only in centers with on-site cardiac surgery (CS). However, rapidly evolving TAVI technology and increasing operator expertise have significantly reduced peri-procedural complications, including those requiring rescue surgery, which occur in less than 0.5% of cases. Furthermore, only a minority of major complications are treated with CS, and the outcomes remain unfavorable. TAVI in centers without CS could represent a solution to reduce waiting times and ensure continuity of care for fragile patients. “TAVI at Home” is a single-arm prospective interventional study. According to sample size calculations based on literature data, the study aims to enroll a total of 200 patients, beginning with a run-in phase of 20 patients to establish safety. The primary endpoint is 30-day all-cause mortality. Secondary endpoints include technical success and the evaluation of single complications 30 days after the procedure. Hospitals without CS that are eligible to perform TAVI must have a high volume of coronary percutaneous interventions, operators with established TAVI experience, collaboration with vascular surgeons, and regular Heart Team meetings to ensure rigorous patient selection.

## 1. Introduction

Aortic stenosis (AS) is the most common valve disease in industrialized countries [1], affecting approximately 5% of adults over the age of 65 years [2], and it is invariably progressive. If untreated, the survival rate is approximately 50% two years after symptom onset and 3% after 5 years [3]. Although surgical aortic valve replacement (SAVR) reduces symptoms and improves survival in patients with symptomatic AS, it is a Class I recommendation in both European and American guidelines [4,5]. However, SAVR is not performed in approximately 25% of cases due to high surgical risk or significant comorbidities [6]. Transcatheter aortic valve implantation (TAVI) has been developed as a less invasive alternative. Over the last two decades, randomized clinical trials have demonstrated the superiority of TAVI compared to medical therapy in inoperable elderly patients, as well as its non-inferiority compared to SAVR in patients with high to low procedural risk [7,8,9,10,11,12,13,14]. The choice between surgical and transcatheter intervention should be based upon careful evaluation of clinical, anatomical, and procedural elements by the Heart Team (HT), weighing the risks and benefits of each approach for the individual patient [4,5]. In the United States (US), 72991 TAVI procedures were performed in 2019, with an 18% increase compared to the previous year [14]. These data, together with the constant technological improvement in devices, the reduction in peri- and post-procedural complications, and the encouraging results on long-term durability, show that TAVI has become the standard of care for most patients with severe degenerative AS. Healthcare systems should adapt to the raised demand for TAVI interventions in order to avoid increasing waiting times, which translates to a high mortality rate during this period. Indeed, studies performed during the early adoption of TAVI showed that 10–14% of patients died while on the TAVI waiting list [15,16]. The cumulative probability of death and heart failure hospitalization while on the waiting list was 2% and 12% at 80 days, respectively, with a relatively constant rise in event rate with increasing waiting times [17]. A long waiting time for TAVI often leads to deterioration in functional capacity [18,19] and is associated with greater risk in the case of an urgent TAVI procedure [20]. Currently, when a patient with an HT indication for TAVI is admitted to a center without cardiac surgery (CS), there are two main pathways to access the procedure. In the first, the patient is referred to the Heart Valve Center at the time of TAVI. In the second, the procedure can be carried out “in service” in a center equipped with CS by expert operators from the spoke center, who follow the patient throughout the entire process. In both cases, the number of TAVI procedures is limited by the Heart Valve Center’s capacity, leading to an increased waiting list. As an alternative, the procedure can also be performed in hospitals without on-site CS, a condition that represents a paradigm shift in the healthcare system organization of many countries. However, rapidly evolving TAVI technology and increasing operator expertise translate into significantly reduced peri-procedural complications, including emergent cardiac surgery (ECS). As reported in Table 1, in the principal National Registries, there has been a decline in structural complications requiring ECS [14,21,22,23,24], the rate of which is actually less than 0.5%. Furthermore, only a minority of major complications are treated with CS, and the outcome of these patients remains unfavorable, even in an optimal setting comprising on-site CS. As reported in the SOURCE registry, patients undergoing ECS have a mortality rate of 52% at 30 days [22]. In a European multicenter study, of 27760 transfemoral TAVI patients, 212 (0.76%) required ECS. The mortality in this group was 46% during the index hospitalization and 78% at one year [25]. The worst prognosis after ECS was also confirmed in a US study that included 47546 consecutive TAVI patients. In this registry, the in-hospital and 30-day all-cause mortality rate in the surgical bailout group was approximately 50%, which was >10-fold higher than in patients who did not require ECS [26]. In very recent study, including 24,010 patients undergoing TAVR between 2007 and 2022, a 30-day mortality rate of 52% was reported [27]. To support the hypothesis that TAVI without on-site CS is not inferior to TAVI performed in CS hospitals, we performed a meta-analysis of the available studies. The results included 21,173 TAVR patients and indicated that the absence of on-site CS does not increase the risk of early death [28].

The aim of the “TAVI at Home” study is to investigate the feasibility and safety of TAVI in centers without on-site CS, in selected patient populations defined by strict inclusion and exclusion criteria.

## 2. Methods and Analysis

### Study Design

“TAVI at Home” is a single-arm, interventional, multicenter, non-profit study conducted on patients with symptomatic and severe AS with an indication for TAVI through transfemoral arterial access in centers without on-site CS. The study is based on a single-arm design, aiming to evaluate the feasibility and safety of this strategy. The robustness of the data will be strengthened through a multicenter approach. The study was approved by the ethics committee of the Local Health Agency of Romagna (C.E.-ROM) and registered on ClinicalTrials.gov (NCT05886517) on the 2 of June 2023. Indication for TAVI will be given by the HT according to inclusion and exclusion criteria (Table 2). The cardiac surgeon is an important part of the HT; in particular, they are fully involved in the clinical and technical evaluation for proper patient selection. Surgical risk stratification will be performed on all patients using LogEuroSCORE, EuroSCORE II, and STS models for subgroup analysis. Eligibility will be clearly stated on the candidate’s personal record. The cardiac surgeon is requested to provide their opinion on the futility of ECS in the case of severe structural complications. A dedicated clinical interview, involving the clinical cardiologist, the interventional cardiologist, and the cardiac surgeon, will be planned to inform patients about their TAVI indication, the possibility of adhering to the experimental study, and to obtain a signed informed consent form. During the clinical interview, it will be clearly explained to the patient that in case of severe structural complications requiring ECS, the intervention will be deemed futile. 

The study will consist of two phases. A pilot phase enrolling 20 patients (10% of the total expected sample) will be conducted to assess the safety of the procedure. In this phase, a maximum of 4 adverse primary outcome events will be allowed based on the range of variability according to previous data [7,8,29,30].

The pilot phase of this study will be conducted at the Morgagni Hospital, Forlì. Once completed, an interim analysis will be performed by an independent data safety monitoring board. In the second phase, up to 10 centers will be involved to reach the final population of 200 patients. 

The study will be performed according to the Declaration of Helsinki and ICH Good Clinical Practice.

AUSL Romagna will be the study sponsor/promoter and the Istituto Romagnolo per lo Studio dei Tumori (IRST) “Dino Amadori” will be the research partner for the study conduction. Centralized and on-site monitoring of the participating centers is planned to ensure patients’ rights and higher quality of the study results.

## 3. Study Population

**Figure 1:**
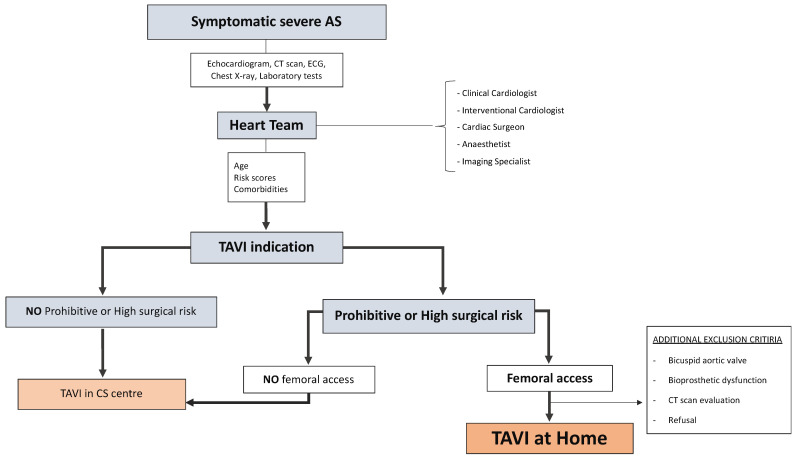
Study scheme.

The study population will consists of patients over 75 years of age with severe symptomatic AS with an indication for TAVI. Eligibility will be determined based on a multidisciplinary discussion within the HT, an assessment of the prohibitive surgical risk according to the VARC consensus, or high surgical risk calculated by specific scores [31,32]. The exclusion criteria are contraindications for transfemoral access, aortic valve bioprosthetic dysfunction, bicuspid aortic valve, and instrumental characteristics, evaluated using CT angiography and associated with an increased risk of major complications. All inclusion and exclusion criteria are listed in detail in Table 2. Patients not fulfilling the inclusion criteria or meeting any exclusion criteria will follow the usual organizational pathway, thus undergoing TAVI in a center with CS. Figure 1 shows the study design.

### 3.1. Characteristics of the Centers

Eligible centers must have regular 24 h interventional cardiology activity [33]. The centers must be part of a network where hospitals refer patients to CS centers according to a hub-and-spoke model, in which patients are promptly transferred for ECS in the event of complications. In this model, collaboration between centers with and without on-site CS is crucial for proper patient evaluation and management.

The general requirements are as follows:

- An overall procedural volume of at least > 36 TAVI/year (3 TAVI/month preferably > 5 TAVI/month) performed in Heart Valve Centers.
- At least two catheterization laboratory rooms, one of which is preferably hybrid.
- At least 1000 coronary angiography and 400 percutaneous coronary angioplasty (PCI) procedures per year.
- Expertise in percutaneous balloon aortic valvuloplasty procedures.
- Expertise in electrophysiology, particularly permanent pacemaker implantation.
- A third-level intensive care unit.
- A vascular surgery unit on site.

### 3.2. Characteristics of the TAVI Operators

There must be at least one expert interventional cardiologist who has performed ≥ 30 TAVI/year as a first operator for at least 3 years and with regular experience in coronary revascularization procedures (more than 75 PCI/year), the use of endovascular devices, and percutaneous treatment of peripheral complications [33].

### 3.3. Sample Size

The sample size was calculated on the basis of previous studies. In the PARTNER cohort B trial, the patients were not considered to be suitable candidates for surgery after evaluation by a cardiologist and two cardiovascular surgeons. Patients that did not have significant comorbidities were enrolled as having prohibitive surgical risk if their STS score was 10% or higher. In this trial, the 30-day all-cause mortality in TAVI patients with prohibitive surgical risk (n = 179) was 5% [7]. In PARTNER cohort A, including 348 high-surgical-risk patients treated with TAVI, the rate of death from any cause was 3.4% at 30 days [8].

In the national US registry, 7710 patients were enrolled, including 1559 (20%) inoperable patients and 6151 (80%) with high-risk cases. Among the 3133 patients for whom 30-day outcome data were available, the incidence of death was 7.6% [29].

In an analysis of the prospective Austrian TAVI registry, 30-day mortality in high-risk and inoperable patients undergoing transfemoral TAVI was 6.2% in patients treated in hospitals with CS on site [30]. Unfortunately, updated 30-day mortality data for patients with prohibitive or high surgical risk are not available. The available literature data are summarized in Table 3.

According to these data, the mean 30-day mortality rate is 6.6%. The sample size will be 200 patients, which demonstrates the non-inferiority of the alternative treatment strategy compared to the standard of care with an alpha error of 5%. We considered a non-inferiority margin of 3%, such as to exclude mortality rates greater than 10% at the upper limit of the 90% confidence interval.

### 3.4. Data Analysis

All of the variables will be described through descriptive statistics: absolute frequencies and percentages for the qualitative and categorical variables, mean and standard deviation for the quantitative variables with normal distribution, and median and interquartile range for the ordinal or quantitative variables with non-normal distribution. For all parameters, 95% confidence intervals will be calculated. Kaplan–Meier curves, the log-rank test, and Cox regression will be performed and used to identify prognostic factors associated with survival. Other parametric (Student’s *t*-test) and non-parametric (chi-square test and Mann–Whitney tests) statistical tests will be used to study the relationship between patient characteristics and secondary endpoints. The significance threshold used for all tests will be 0.05. All analyses will be performed using STATA software, version 17.0.

### 3.5. Outcome Measures

The primary endpoint will be 30-day all-cause mortality, which will be measured as the number of deaths within 30 days of the procedure. Secondary endpoints will include the assessment of technical success and the evaluation of single complications at 30 days after the procedure [32]. Technical success will be defined according to VARC-3 consensus [32]:

- Freedom from mortality.
- Successful access, delivery of devices, and retrieval of the delivery system.
- Correct positioning of a single prosthetic heart valve into the proper anatomical location.
- Freedom from surgery or intervention related to the device (excluding pacemaker implantation) or to a major vascular, access-related, or cardiac structural complication.

## 4. Conclusions

The aim of this study is to evaluate an innovative treatment strategy for patients with severe AS and indications for TAVI. The results are expected to provide prospective evidence on the efficacy and safety of TAVI performed in centers without on-site CS. In addition, extending TAVI procedures to centers without CS could represent a solution to reduce waiting times and ensure continuity of care, particularly for elderly and frail patients, for whom emergency cardiac surgery in the case of severe complications may be futile.

## Figures and Tables

**Table 1:** Conversion to emergent cardiac surgery and principal structural complications after TAVI in principal international registries [14,21,22,23,24]. # 30-day mortality; * in-hospital mortality; ^ 24 h mortality.

	Source (2007–2009)	Source 3 (2014–2015)	France 2 (2010–2012)	France (2013–2015)	Gary (2010–2015)	STS (2011–2019)
N.° of patients	1038	1947	4165	12,557	15,964	276,316
Mean age	81	81	83	83	81	81
LogES (%)	27	18	22	18	18	-
Death (%)	8.5 #	2.2 #	8.1 *	4.4 *	1 ^/5.2 *	2.0 */3.1 #
Conversion to open surgery (%)	2.7	0.6	1.2	0.5	1.3	0.6
Ring rapture (%)	-	0.2	0.3	0.4	0.4	-
Aortic dissection (%)	-	-	0.2	0.4	0.2	-
Coronary obstruction (%)	-	0.4	-	-	-	-
Cardiac tamponade (%)	-	-	1.3	2	1.0	-
Valve malposition (%)	0.3	-	1.3	1.1	-	-
2° Valve implantation (%)	-	0.7	2.3	1.8	-	-

**Table 2:** Eligibility criteria.

Inclusion criteria - Severe symptomatic aortic valve stenosis - Age ≥ 75 years - Patients evaluated by Heart Team and selected on the basis of at least one of the following criteria: 1. High surgical risk (LogES > 20%, EuroSCORE II > 8%, and STS score > 8%) 2. Porcelain aorta 3. Hostile chest 4. Severe fragility 5. Severe liver disease/cirrhosis 6. Presence of a patent graft of an internal mammary artery crossing midline and/or adherent to the posterior table of the sternum 7. Severe pulmonary hypertension or severe right ventricular dysfunction - Feasible transfemoral access - Signed informed consent form
Exclusion criteria - Aortic valve bioprosthetic dysfunction - Bicuspid aortic valve - Anatomical features associated with an increased risk of major complications: 1. Severe left ventricle outflow tract or sub-annular calcification 2. Valvular ring to coronary ostia distance < 10 mm 3. Severe aortic root dilatation or out-of-range aortic annulus diameters for TAVI - Contraindication for femoral access

**Table 3:** Thirty-day all-cause mortality after TAVI in patients with high or prohibitive surgical risk [7,8,29,30].

	Partner Trial		STS/ACCTVT Registry	Austrian Registry
	Inoperable	High-Risk	Inoperable + High-Risk	Inoperable + High-Risk
30 Days	(9/179) 5.0%	(12/348) 3.4%	(239/3133) 7.6%	(2/290) 6.9%

## Data Availability

No new data will be created or analyzed for this article and data sharing will not be applicable. The raw data that will be generated in the course of the clinical study will be made available by the authors upon request.

## Abbreviations

Aortic stenosis (AS); cardiac surgery (CS); Heart Team (HT); transcatheter aortic valve implantation (TAVI); surgical aortic valve replacement (SAVR); United States (US).
